# The Role of Echocardiography in the Diagnosis and Prognosis of Pulmonary Hypertension

**DOI:** 10.3390/jpm14050474

**Published:** 2024-04-29

**Authors:** Nikolaos P. E. Kadoglou, Elina Khattab, Nikolaos Velidakis, Evaggelia Gkougkoudi, Michael M. Myrianthefs

**Affiliations:** 1 Medical School, University of Cyprus, 215/6 Old Road Lefkosias-Lemesou, Aglatzia, Nicosia 2029, Cyprus; khattab.elina@ucy.ac.cy (E.K.); velidakis.nik@gmail.com (N.V.); gkougkoudi.evangelia@ucy.ac.cy (E.G.); 2 Cardiology Department, Nicosia General Hospital, Lemesou 215, Strovolos, Nicosia 2029, Cyprus; myr.michael@shso.org.cy

**Keywords:** pulmonary hypertension, echocardiography, pulmonary arterial systolic pressure (PASP), diagnosis, prognosis, speckle tracking

## Abstract

The right heart catheterisation constitutes the gold standard for pulmonary hypertension (PH) diagnosis. However, echocardiography remains a reliable, non-invasive, inexpensive, convenient, and easily reproducible modality not only for the preliminary screening of PH but also for PH prognosis. The aim of this review is to describe a cluster of echocardiographic parameters for the detection and prognosis of PH and analyse the challenges of echocardiography implementation in patients with suspected or established PH. The most important echocardiographic index is the calculation of pulmonary arterial systolic pressure (PASP) through the tricuspid regurgitation (TR). It has shown high correlation with invasive measurement of pulmonary pressure, but several drawbacks have questioned its accuracy. Besides this, the right ventricular outflow track acceleration time (RVOT-AT) has been proposed for PH diagnosis. A plethora of echocardiographic indices: right atrial area, pericardial effusion, the tricuspid annular plane systolic excursion (TAPSE), the TAPSE/PASP ratio, tricuspid annular systolic velocity (s′), can reflect the severity and prognosis of PH. Recent advances in echocardiography with 3-dimensional right ventricular (RV) ejection fraction, RV free wall strain and right atrial strain may further assist the prognosis of PH.

## 1. Introduction

Pulmonary hypertension (PH) is a multi-faceted, lethal condition characterized by increased pulmonary artery pressure, which may be attributed to a long list of conditions [1,2]. According to the most recent guidelines of the European Society of Cardiology (ESC) PH is defined as a mean pulmonary artery pressure (mPAP) above 20 mmHg, using the right heart catheterization (RHC) at rest [3]. Timely diagnosis, evaluation, and treatment are particularly important in patients with PH. RHC remains the gold standard technique for PH diagnosis, but due to its invasive nature it is difficult to perform or repeat in daily clinical practice. Alternatively, rest echocardiography is widely used for initial screening for PH, because it is an easy, repeatable, inexpensive technique and its measurements show high correlation with RHC and unravel progressive right ventricular (RV) dysfunction. Echocardiography mainly calculates pulmonary arterial systolic pressure (PASP) and draws the probability of PH presence [4]. However, its diagnostic and prognostic role remains challenging due to a major limitation: its measurements rely on assumptions while altered hemodynamic conditions (such as severe tricuspid regurgitation; TR) may compromise the accuracy of echocardiographic parameters. Regarding the impact of PH on right cavities and their function, additional classical echocardiographic parameters, like the right atrial area (RAA), tricuspid annular plane systolic excursion (TAPSE) have been proposed to describe the severity of PH [5].

Besides diagnosis, the prognostic value of several echocardiographic parameters is wondering. Several systematic reviews and meta-analyses have been conducted regarding the role of echocardiography in the severity and prognosis of PH [6,7]. Pericardial effusion (PE), RAA and TAPSE have been proposed as prognostic factors. Novel echocardiographic parameters like 3-dimension (3D) echocardiography, RV speckle tracking, right atrium (RA) strain have emerged as more accurate techniques with increasing prognostic power among patients with PH. Nevertheless, the echocardiographic assessment of PH and its consequences must address important challenges due to required assumptions and inherent pitfalls. The operator should always be aware of them before getting firm conclusions on the prediction of PH progression.

The objective of this review is to delineate the key echocardiographic parameters proposed for detecting and prognosticating PH. A comprehensive examination of the challenges confronting echocardiography in patients with suspected or confirmed PH could facilitate its effective utilization.

## 2. Materials and Methods

We conducted a literature search in the English language for publications in the MEDLINE and EMBASE, Web of Science, Cochrane, and Google Scholar databases from 1990 to February 2024. The following search terms, for titles and abstracts, including Medical Subject Headings (MeSH), were used: pulmonary hypertension; echocardiography; pulmonary arterial systolic pressure (PASP); diagnosis; prognosis; speckle tracking. Three investigators (EG, NV and EK) independently performed the literature search. We included experimental studies, in vitro and in vivo, and clinical studies as well. We further limited our literature search by setting the following exclusion criteria: studies with full text unavailable, published in languages other than English, and conference abstracts. The reference lists of the identified articles were checked for any additional relevant articles, especially among reviews.

## 3. Classic Echocardiographic Parameters: Diagnosis

### 3.1. Studies Comparing Echocardiography with Right Heart Catheterization (RHC)

The application of echocardiography in the diagnostic realm of PH has gained large recognition for the past decade. Echocardiography is an easy, inexpensive, easily available, uncomplicated, and repeatable technique compared to RHC. A variety of metrics have been proposed for this objective, adding to the ongoing debate on the agreement between non-invasive (echocardiography) and invasive assessment (RHC) of pulmonary pressure. The current body of evidence highlights a significant level of concurrence, yet discernible discrepancies persist in certain facets of PH, underscoring the necessity for a comprehensive review to reconcile the variations among diverse sources. Forty years ago, the first published study indicated a high correlation between invasive and non-invasive methods [8]. Regarding the RHC as the gold-standard technique for pulmonary artery pressure measurement and PH diagnosis, numerous studies have been conducted to validate the accuracy of echocardiography in pulmonary pressure estimation, presenting high correlation coefficients of 0.97, but with limited number of participants in each study [9,10,11,12,13]. A retrospective analysis of 1695 individuals undergoing both RHC and echocardiography, with a maximum time interval of five days, aimed to investigate the correlation between invasive and echocardiographic measurements of PASP and mPAP [14]. High Pearson’s correlation coefficients were identified between measurements from the two imaging modalities (*r* = 0.87, *r* = 0.82 respectively; *p*-value < 0.001 for both coefficients), indicating high correlation between them [14]. High correlation coefficients have been identified by other studies, using similar methodology but smaller number of participants [15]. One of the largest and most recent studies also identified a high correlation between the invasive and non-invasive method for PASP in a population of 1400 individuals with severe aortic stenosis [16]. On the other hand, there are studies exhibiting lower degree of correlation, especially when individuals with lung diseases are included [17,18,19]. Consequently, a significant limitation of echocardiography arises from the propensity for overdiagnosing PH in populations afflicted with established lung diseases, such as chronic obstructive pulmonary disease or pulmonary interstitial disease [20]. Summarizing the above results, a recent systematic review investigated the accuracy of echocardiography in the detection of PH in comparison to RHC [7]. Overall, 17 studies with over 3656 participants, 1342 of whom had a confirmed diagnosis of PH, were included in the analysis. All the included studies compared echocardiography versus RHC to test their sensitivity in PH diagnosis, setting the mean PAP > 25 mmHg as the diagnostic threshold. The time interval between RHC and echocardiography was less than seven days, to limit bias in the systematic review. There was great heterogeneity between studies regarding the PASP threshold used for the diagnosis of PH through echocardiography, which ranged from 30 to 47 mmHg. After analysis, the median value of sensitivity of echocardiography was 87% (range: 40–98%) and the median value of its specificity was 86% (range: 33–100%). A previous meta-analysis of 29 studies indicated a pooled correlation coefficient of 0.70 (95% CI: 0.67, 0.73), and again a high heterogeneity was observed between the included studies, making it challenging to draw definitive and reliable conclusions [21].

The correlation between these two modalities may be influenced by the operator’s level of experience, given that echocardiography is an operator-dependent technique with inherent limitations in diagnostic accuracy [15]. The implementation of semi-automated measurement of the RA pressure (RAP) may attenuate the impact of operator’s subjectivity [22]. Various echocardiographic measurements are employed, each possessing its distinct set of advantages and disadvantages. They are depicted in Table 1 and analyzed in the following sections:

### 3.2. Tricuspid Regurgitation Velocity Peak (TRVpeak) and PASP Calculation

The calculation of PASP through Doppler echocardiography is indirect and relies on the measurement of RV-RA pressure gradient and the assumption of RAP value. This is the most widely used approach for the quantification of PASP in echocardiography. In particular, the echocardiographer measures the peak velocity of TR (TRVpeak) and then using the Bernoulli equation can calculate the pressure gradient of RV-RA = 4Vpeak2. After adding an estimate of RAP, normally ranging 0–5 mmHg, to RV-RA pressure gradient, the echocardiographer gets the estimated PASP. RAP assumption is based on the size of inferior vena cava (IVC) and its collapsibility during inspiration [23]. The PASP estimation from echocardiography seems easy, repeatable, immediately available, and reproducible compared to the gold-standard measurements with RHC. Previous studies have supported the high accuracy of echocardiography in calculation of PASP compared to RHC. One significant limitation of echocardiography, however, lies in the indirect measurement of PASP, which depends on estimating the RV-RA pressure gradient and assumes a specific RAP value. Also, the required assumptions have raised concerns about the degree of agreement between those techniques in pulmonary pressures calculation in specific subpopulations.

In the 2022 guidelines for the diagnosis and treatment of PH, the ESC and the European Respiratory Society (ERS) advise the use of TRVpeak as the main index of PH [3]. According to these guidelines, TRVpeak above 2.8 m/s is suggestive of PH but the sole use of TRVpeak is insufficient for determining the true pressure gradients and possible the presence of PH. In severe TR, the peak velocity may underestimate pressure gradients, while in patients with high cardiac output due to liver disease or sickle cell disease or in case of artefacts, the pressure gradients may be overestimated [24]. These limitations underscore the reasons why current guidelines emphasize assigning probabilities based on a cluster of echocardiographic measurements, rather than relying on them for a definitive diagnosis.

A recent clinical trial investigated among others the correlation between invasive and echocardiographic measurements of PASP in a population of 243 patients with severe TR [25]. The threshold of 50 mmHg was used for both methods to classify an individual as PH positive. The time interval between the two methods varied from two to 17 days, with a median time the six days. This analysis indicated a moderate correlation between invasive and non-invasive PASP (*r* = 0.47; *p*-value < 0.01). Simultaneously, the sensitivity and specificity of echocardiography for the RHC-diagnosed PH was 55% and 74% respectively. This is attributed partially to the rapid equalization of pressures between RA and RV, resulting in a practically low RV-RA pressure gradient, consequently to the underestimation of RAP [23,25]. Interestingly, in the study, individuals with a false negative diagnosis of PH based on echocardiography had the more severe grades of TR [25]. Those inconsistencies in results do not restrict the wide use of echocardiography in the diagnosis of PH but stress the importance of a more careful interpretation in patients with severe TR and the need for more studies to identify how echocardiographic measurements may apply.

An umbrella meta-analysis documented the high sensitivity of echocardiography-based PASP calculation to diagnose PH [26]. However, the included studies showed a high heterogeneity, attributed mainly to the different ways of PASP calculation. Most of them used TRVpeak, while the rest used the jugular vein pressure. The discrepancy between echocardiography and RHC to calculate both mPAP and PASP was also outlined in a recent, small, comparative study, due to the overestimation of mPAP and PASP by echocardiography [27]. Females, individuals with arrhythmias or patients under diuretic therapy are more susceptible to this overestimation. Another significant explanation for the discordance between echocardiography and RHC is the measurement of different parameters PASP and mPAP, respectively. The latter is not usually obtained in echocardiography and some studies have proposed the non-invasively measured PASP with a cut-off value > 40 mmHg showing the highest sensitivity and specificity for PH diagnosis [14,15,16].

Although IVC dimensions can be accurately measured in most cases from the sub-costal view, it is not always a sensitive and representative index of RAP [23]. For example, the IVC may be permanently distended in patients with heart or kidney failure due to chronic exposure to volume overload. Thereby, the IVC remains constantly enlarged, even in euvolemic patients, and it is dissociated from volume changes and RAP assumption. In healthy, young athletes, the IVC may be normally dilated and may not reflect an increased RAP [23]. Additionally, in ventilator-dependent individuals, the reduced IVC collapse may reduce the accuracy of RAP estimation. In general, the reliability of IVC collapse is diminished in intermediate-to-high values of RAP. Alternative parameters can be considered, like a tricuspid E/E’ ratio > 6 and diastolic flow predominance in the hepatic veins, when a restrictive right-sided diastolic filling pattern is recorded [23]. The latter parameters require further investigation.

### 3.3. Right Ventricular Outflow Track Acceleration Time (RVOT-AT)

The right ventricular outflow track (RVOT) acceleration time (RVOT-AT) was firstly introduced 40 years ago and reflects the time between the start of right ventricular ejection and the peak flow velocity across the right ventricular outflow track, just below the pulmonary valve [28]. According to this concept, the flow of blood in the RVOT accelerates more, in individuals with PH compared to those with normal pulmonary pressures. As a result, RVOT-AT shortens when pulmonary pressures increase. According to the latest guidelines, values under the cut-off of 105 ms are suggestive of pre-capillary PH but this needs to be assessed in combination with other parameters [3]. Previous studies have demonstrated the strong relationship of RVOT-AT with PASP, independent of TR severity [29]. According to a recent meta-analysis RVOT-AT was highly correlated with PASP values [26]. Another meta-analysis of 21 studies and 1280 patients showed adequately high pooled sensitivity [0.84 (95% CI: 0.75, 0.90)] and specificity [0.84 (95% CI: 0.78, 0.89)] of RVOT-AT to diagnose PH [30]. The sensitivity of included studies showed high variability, while the heterogeneity for specificity was lower, indicating more consistent results. This can be partly explained by the different cut-off values used in the included studies, ranging from 90 to 104 ms, showing an inverse relationship between cut-off value and sensitivity at the expense of lower specificity.

Regarding co-founders, RVOT-AT can be easily obtained in most individuals, its accuracy seems not to be influenced by the etiology of PH, but the presence of arrythmias may affect its sensitivity. Moreover, the placement of sample volume at the RVOT may challenge the results. It seems that the right measurement of pulmonary acceleration time below 100 ms increases the probability of PH by nearly 5 times (OR: 4.8; 95% CI: 1.1, 20.4; *p*: 0.34) [31]. Unlike to the data from meta-analyses, an observational study with 236 participants admitted in the intensive care unit for acute cardiovascular or respiratory failure indicated a poor inverse correlation between PASP measurements through Doppler echocardiography and RVOT-AT [32]. It seems that the impaired RV function significantly weakens the correlation of RVOT-AT with PASP in cardiothoracic critically ill patients, but this requires further validation.

### 3.4. Pulmonary Regurgitation

According to the recent guidelines, the early diastolic pulmonary regurgitation velocity is suggested as one of the parameters that needs to be considered when there is a suspicion of PH [3]. More precisely, values over 2.2 m/s are among the factors that can help to define the probability of PH. More detailed data are required because it is an uncommon finding among patients with PH and therefore cannot be used as a screening parameter of PH.

### 3.5. The Potential Roles of the Echocardiography in the Differential Diagnosis of Pulmonary Hypertension

Distinguishing between pre- and postcapillary PH poses a persistent challenge, necessitating invasive assessment through RHC [33]. In cases of PH due to left heart disease, echocardiography allows the identification of mitral or aortic valvulopathy, chronic ischemic heart disease, and LV diastolic or systolic dysfunction. This imaging modality is capable of capturing significant changes in the structure, size, and function of the heart [33]. Moreover, the type of remodeling of the heart varies depending on the form of PH (pre- or post-capillary) and aids in understanding the pathophysiology of the underlying disease. In this scenario, there is a clear predominance of the left chambers, with LA and/or LV dilatation, LV hypertrophy, reduced LV ejection fraction, eventual regional LV hypokinesis or akinesis, and the presence of calcifications with altered mitral and/or aortic valve function [33]. In pulmonary arterial hypertension (PAH), echocardiography may reveal a predominance of the right heart chambers, such as an RV-forming apex, RV hypertrophy, and systolic dysfunction. Additionally, it may indicate IVC dilatation, notched pulsed-wave Doppler in the RVOT, and often an increase in tricuspid and pulmonary regurgitant jets [33]. Proposing a method to distinguish between pre- and post-capillary pulmonary hypertension (PH), several studies [34,35,36] have introduced a multiparametric echo score. The Opotowsky score, incorporates easily measurable and reproducible echocardiographic parameters, including the E/e’ ratio, dimensions of the LA, and RVOT pulsed-wave Doppler mid-systolic notch [34]. Additionally, the D’Alto score assesses the dimensions of the right versus left heart chambers, the RV forming the heart apex at end-diastole, LV eccentricity index measured at the mid-papillary level at end-diastole in the short-axis view, IVC diameter and collapsibility, and E/e’ ratio [35,36]. In patients with combined pre- and post-capillary PH (cpc-PH), a condition frequently observed in mitral stenosis or HF with preserved EF, both left and right heart chambers may concurrently dilate due to overload. Additionally, features such as LV D-shape and RV hypertrophy may be evident.

## 4. Classic Echocardiographic Parameters: Prognosis

**Figure 1:**
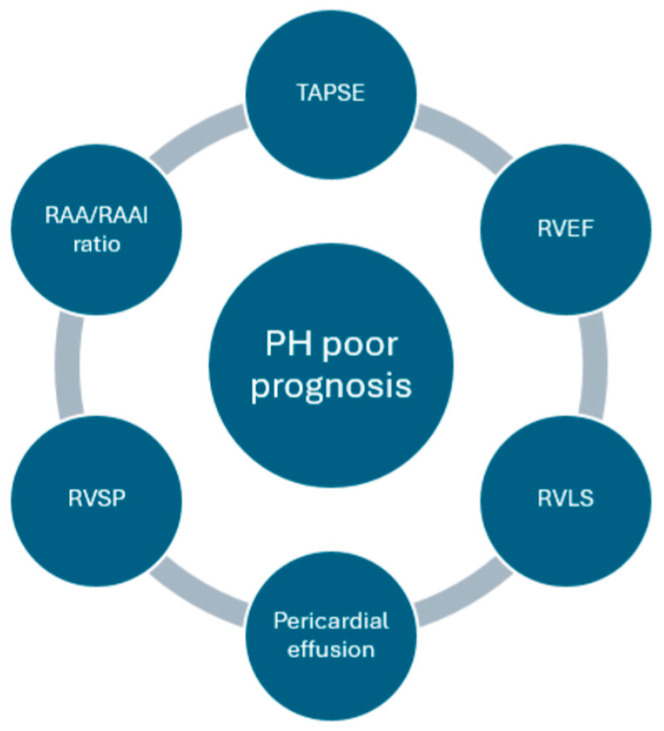
Echocardiographic parameters related to pulmonary hypertension prognosis.

PH is linked to poor prognosis, with an estimated 1-year mortality rate exceeding 20%, when is accompanied by signs of RV failure, rapid symptom deterioration, recurrent syncope episodes, and elevated levels of B-type natriuretic peptide or N-terminal pro-B-type natriuretic peptide surpassing 800 ng/L and 1100 ng/L, respectively [3]. Irrespective of etiology, the survival rate of patients with PH exhibited a gradual decline over the years, reaching approximately 50% at the seven-year follow-up from the initial diagnosis [37,38]. A meta-analysis of 15 studies demonstrated that even mild PH was associated with 19% elevated risk of mortality over 5 years [39]. The prognosis of PH is significantly influenced by the early diagnosis and prompt initiation of treatment [37,40]. Echocardiography in addition to its diagnostic value when PH is suspected, remains a crucial prognostic tool, by detecting preclinical stages of the disease, exploring treatment options, and monitoring the effectiveness of specific therapies [41,42]. Figure 1 depicts the most important echocardiographic indices of PH prognosis.

### 4.1. Tricuspid Annular Plane Systolic Excursion (TAPSE)

**Figure 2:**
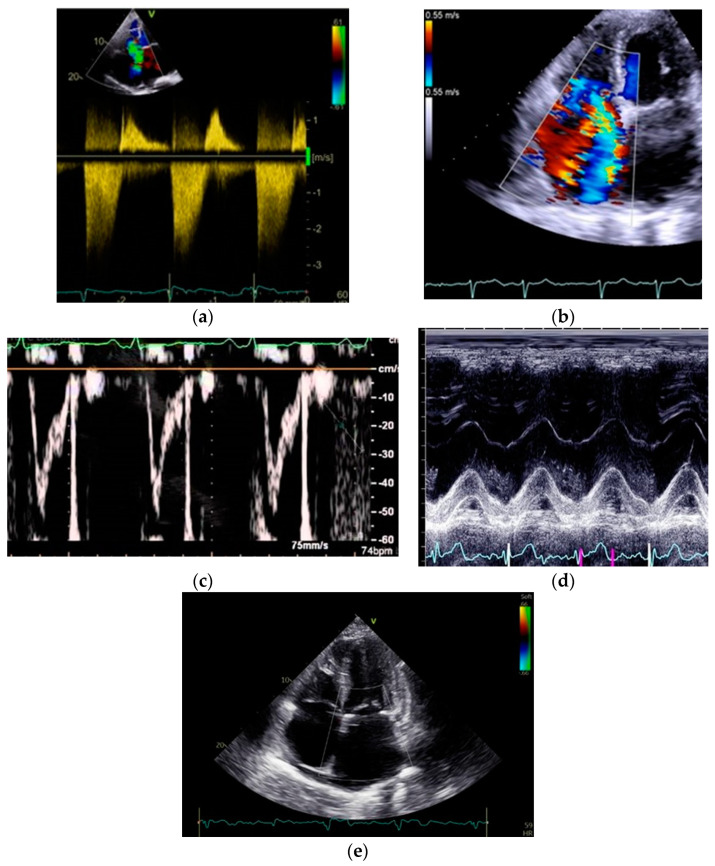
Classical echocardiographic indices of pulmonary hypertension diagnosis and prognosis: (**a**) TRVpeak doppler; (**b**) TR color doppler; (**c**) RVOT-AT; (**d**) TAPSE; (**e**) Pericardial effusion and RA enlargement.

TAPSE has emerged as a crucial prognostic indicator in PH [43,44,45,46], particularly in patients with idiopathic PH, systemic sclerosis-associated PH, and Eisenmenger’s syndrome. Shukla M, et al., suggested that TAPSE plays a crucial role as a prognostic indicator in patients with PH, correlating with higher all-cause mortality [47]. Nonetheless, it is worth noting that TAPSE is influenced by the angle of measurement and operator-dependent factors. Variations may occur based on heart motion, the severity of TR, and the dynamic interaction between the RV and RA [48]. In a recent study involving children with PH, no significant correlation was observed between TAPSE and transplant-free survival. However, the combination of RA size and left ventricular (LV) eccentricity index emerged as a robust prognostic marker for transplant-free survival, potentially complemented by TAPSE [46]. The combined assessment of TAPSE with other indices may overcome those shortcomings. TAPSE/PASP ratio serves as a non-invasive metric for assessing RV-arterial coupling and is influenced by RV diastolic stiffness, particularly in cases of severe PH [49]. The TAPSE/PASP ratio is highlighted as a significant prognostic factor in the assessment of PH, with thresholds of 0.32 mm/mmHg and 0.19 mm/mmHg, distinguishing high, intermediate, and low mortality risk, respectively, as acknowledged in the 2022 ESC guidelines [3,26,49]. A retrospective study of 677 patients with PAH demonstrated that improvement in the TAPSE/PASP ratio under targeted therapies is associated with a low-risk status and a significant reduction in PVR [50]. Moreover, one of the biggest challenges in echocardiography is to predict the RV systolic reserve and its response to increase afterload. This is important in clinical decision making in patients with severe TR and concomitant PH, who are candidates for interventional TV correction [51]. Moreover, the RAA exceeding 26 cm^2^ and the TAPSE/PASP ratio falling below 0.19 mm/mmHg, are correlated with heightened mortality (>20% in one year) in patients with PH [3]. Figure 2 illustrates the classical echocardiographic parameters of PH diagnosis and prognosis. 

The tricuspid annular systolic velocity (s′) has long been accompanied by TAPSE measurements. Many studies have supported it among others as a strong index of RV dysfunction with significant prognostic value [52]. Patients planned to undergo surgery of left heart valvular disease and concomitant PH should be always evaluated for TV repair when severe TR is present [52]. This is a great challenge in echocardiography because the assessment of RV systolic function and its response to altered volume and pressure load will influence the efficacy of interventional therapy of TR. The prognostic value of S’ warrants further investigation in larger prospective studies to validate previous findings.

### 4.2. Right Ventricular Size

In patients with PH, RV dilation is usually observed because of increased afterload and preload [52]. At the early stages of PH development, the RV effectively responds to increased afterload in PH by boosting contractility while keeping dimensions intact (homeometric adaptation). Additionally, it resorts to increased dimensions (heterometric adaptation) when the improvement in systolic function is insufficient to uphold RV-arterial coupling [53]. After excluding transient volume overload, the RV dilatation combined with RV dysfunction can be an indirect poor prognostic factor in patients with PH and should always be considered in decision making. In severe PH, RV failure inevitably leads to enlarged RV dimensions. Thus, a therapeutic focus in the management of severe PH should be on reversing RV dimensions back to normal [53]. A prospective study involving 102 PH patients demonstrated that right heart reverse remodeling after 1 year of PH treatment is an independent predictor of prognosis in this population. Also, they showed that the likelihood of reverse remodeling is proportional to decreased PVR [53]. Basal and mid RV diameter as well as RV length can be measured. Basal RV diameter is calculated in the maximal transverse diameter in the basal one third of the RV while mid RV diameter in the level of LV papillary muscles [54]. Values which are considered abnormal are above 41 and 35 mm, respectively [55]. RV length corresponds to the distance between the tricuspid annulus until the RV apex during end diastole and the cut-off value is 83 mm [55]. The cut-off values of RV size remain to be determined, and clarification of their relationship with prognosis is needed [56].

### 4.3. Right Ventricular Function

Impaired RV ejection fraction (RVEF) in RV failure is associated with adverse outcomes in PH, possibly through its connection with RA size and TR mechanism. Fractional area change (FAC), a widely used surrogate of RVEF with values <31–35% indicating RV dysfunction [43,57,58] shows weak correlation with mortality in PH [59]. RV dysfunction frequently dictates the severity of patients’ symptoms and stands as a primary contributor to PH mortality [60,61]. Moreover, the diastolic LV eccentricity index, a consequence of prolonged RV contraction in response to elevated PASP, is evident in severe PH and prognosticates adverse outcomes [46]. Increased RAP, reflecting right heart failure, is a crucial prognostic indicator in PH [60,62], and the echocardiographic parameter A’ (a reflection of atrial contraction) is the most accurate surrogate of catheterization-based RAP [63].

An alternative study proposed that apical traction, a distinct motion pattern of the heart characterized by the cardiac apex being pulled towards the LV, is linked to poorer outcomes in patients with PH [64]. Additionally, a study led by Sano H et al. demonstrated that PH patients exhibiting a RV relative wall thickness < 0.21 and lacking mid-term RV reverse remodeling (characterized by a relative decrease in the RV end-systole area of at least 15%) experienced poorer long-term outcomes following treatment [65].

### 4.4. Right Atrium Size and Function

RA dilation and RAP elevation, resulting from RV dysfunction, TR and fluid overload are important prognostic factors in patients with PH [46,66]. RA size and RAP are indicative of RV overload in PH and are recognized as risk factors for adverse outcomes [67]. RAP is merely essential for PASP quantification, however elevated levels have shown a correlation with increased mortality [68]. A meta-analysis conducted by Liu K, et al. (2020) [45] sought to investigate the role of RAA and RAA index (RAAI). It demonstrated that RAA/RAAI ratio is related with increased risk of poor prognosis in this population, and the risk of all-cause mortality was increased by 50% for every 5 unit increase in RAA/RAAI ratio. Also, RAA increase is associated with mortality and transplantation [53]. Furthermore, the novel RA function index (RAFI), calculated as: RAFI  =  RAEF × RVOT–VTI/RAESVI (RAEF: emptying fraction of right atrium, RVOT-VTI: right ventricular outflow tract velocity-time integral: RA end-systolic volume index), can be a robust predictor of clinical outcomes in individuals with precapillary PH [61,69].

### 4.5. Pericardial Effusion

Additionally, the presence of moderate or large PE is also associated with high mortality rate in this population, while minimal PE is related with an intermediate risk (estimated 1-year mortality rate between 5–20%) [3]. PE is considered a manifestation of RV failure, where fluid accumulation occurs due to elevated RAP, resulting in compromised venous and lymphatic drainage [57]. Moreover, a meta-analysis showed the presence of PE to be independently associated with mortality, transplantation, and clinical deterioration in patients with PH [70].

### 4.6. Right Ventricular Systolic Pressure (RVSP)

RVSP is usually mentioned in published studies as a substitute for PASP. The prognostic significance of estimated RVSP at rest is negligible, showing no correlation with disease progression and exerting no influence on treatment strategies. An increase in RVSP does not necessarily reflect disease deterioration and a decrease in RVSP does not reflect improvement in prognosis [3]. A large retrospective cohort study used echocardiographic data from 47,000 participants, dividing them according to their RVSP values [71]. Participants with mild PH, defined by RVSP 33–39 mmHg, compared to those with RVSP lower than 33 mmHg, had higher mean TAPSE values (2.7 m/s vs. 2.2 m/s respectively), indicating a compensatory increase of RV function. Simultaneously, a higher occurrence of RV dilation and mean RV end diastolic diameter (EDV) was observed in the group of mild PH in comparison to no PH. Another point of consideration is the impaired RV-PA (right ventricle-pulmonary artery) coupling, as expressed by the ratio of TAPSE/RVSP. Furthermore, worse RV function and RV-PA coupling were observed in the PH group, defined by RVSP values above 40 mmHg, compared to both no PH and mild PH group. These results indicate that RSVP values between 33 and 39 mmHg, although considered normal, may be associated with RV dysfunction and dilatation.

### 4.7. Other Echocardiographic Parameters

Another study investigated the predictive value of pulmonary arterial capacitance (PAC) using echocardiographic parameters. The study employed the ratio RVOT-VTI/PASP and demonstrated its association with mortality in patients with PH [72]. Similarly, reduced RV systolic function, as assessed by TAPSE, accompanied with elevated RV thickness were correlated with increased mortality. In contrast, LV systolic function, LV diastolic parameters, PASP, or echocardiography-derived pulmonary vascular resistance (PVR) did not exhibit a significant association with heightened mortality in patients with cardiopulmonary co-morbidities [73].

Another interesting index is the Tei myocardial performance index (MPI), which was studied in patients with chronic thromboembolic PH in comparison to healthy individuals [74]. Right ventricular Tei index is defined as (A—B)/B, where A (IVCT + SC + IVRT) is the period including the isovolumetric contraction time (IVCT), systolic contraction (S), and the isovolumetric relaxation time (IVRT). B is defined as the time of systolic contraction. MPI is calculated from Doppler recordings from the lateral tricuspid annular velocity and represents the RV myocardial function. Individuals with PH had statistically higher values of MPI compared to healthy controls (0.52 vs. 0.27) and exhibit a strong correlation between MPI and PVR. This association is preserved, even after pulmonary thromboendoarterectomy, although slightly attenuated and may assist to predict the progression.

## 5. Novel Echocardiographic Techniques for Diagnosis and Prognosis

### 5.1. RV Function Assessment with 3D Echocardiography

There are specific conditions that are associated with inaccurate measurements of RV dimensions and volumes. For example, PVR can increase when cardiac output cannot be maintained and so RVSP may be constant or even decrease. Similarly, when TR increases, RVSP values may decrease [75]. A way to address this problem is probably through 3D-echoardiography. In a recent study encompassing 96 pediatric patients with PH, noteworthy distinctions emerged in RV EDV index (RV EDVi), RVEF, and free wall RVLS among those with and without clinical adverse events. The findings underscored the prognostic relevance of 3D RV functional indices and volumes in pediatric PH patients [76]. An additional study revealed that 3D echocardiography exhibited substantial agreement with cardiac magnetic resonance imaging, considered the gold standard for the evaluation of RV volumes and function in patients with PH [77]. This encompassed assessments such as EDV, end-systolic volume (ESV), stroke volume (SV), and RVEF. The measurements from both techniques demonstrated a comparable correlation with other clinical prognostic parameters in these patients. Consequently, the utilization of 3D echocardiography holds significance in the clinical evaluation of individuals with PH [77]. Data from PVR seems to positively correlate with RVESV index and negatively correlate with LVEDV) index in small scale study. Additionally, mPAP had a positive correlation with both RVEDV index and RVESV index. Two further indices were calculated as the ratios of RVEDV/LVEDV index and RVESV/LVESV index, which represent the characteristic pattern of enlarged RV and compressed LV in PH. Both indices had positive linear associations with PVR (*r* = 0.67; *r* = 0.55) and may weakly support the PH diagnosis prognosis.

### 5.2. Right Ventricle Speckle Tracking

Another imaging technique that is investigated for its possible use in PH diagnosis is 2D speckle-tracking echocardiography (2D-STE) [78]. Through 2D-STE several measurements regarding RV function can be made including peak systolic strain (PSS) and post-systolic index (PSI) of the RV free wall. Results from a small observational study indicated the cut-off value of −20.75% for PSS with high sensitivity and specificity for detecting mPAP above 35 mmHg (87.5%, 87.5% respectively). The same cut-off value offers even higher sensitivity and specificity for a detection of PVR above 5 Wood units. Furthermore, PSS seems to be the only independent factor for elevated PVR and mPAP. Another measurement that can be extracted through 2D-STE is RV peak strain (RV-PS). Its absolute value seems to be lower in PH patients in comparison to those without PH and having a high correlation with mPAP [79]. It needs to be stressed that this study was conducted on a population with chronic thromboembolic PH, perhaps limiting the generalizability of the results.

Indicators of RV myocardial deformation, such as RV strain and strain rate index, are pivotal in gauging the severity of PH, but also for prognosis. Based on accumulated evidence, a RVLS evaluation (≥−19%) stands out as a crucial predictor of all-cause mortality [37,62,80,81] and its prognostic significance has been extensively highlighted [26]. Even more, RV strain above −15% (both global and free wall in absolute values) serves as an indicator of the poorer survival [60]. A recent umbrella review of 13 meta-analyses evaluated the prognostic role of echocardiographic parameters in PH. RVLS in addition to another 4 parameters (PE, RAA, RAA index, TAPSE) correlated with PH prognosis [26]. In two meta-analyses assessing the prognostic implications of RVLS in PH patients, the results demonstrated that patients with a 22% reduction in RVLS exhibited a markedly elevated risk of all-cause mortality. Also, RVLS proved to be superior index of mortality than TAPSE [47,82]. RVLS has proven to be a reliable predictor of both outcome and responsiveness to medical therapy in PH [59]. However, in patients with PH and Eisenmenger syndrome RV transverse strain has a higher predictive value than RVLS [83].

### 5.3. Right Atrium Volume and Strain

Another study illustrated that 3D echocardiography enables the measurement of RA volume and phasic function, highlighting its feasibility and consistency throughout the cardiac cycle [84]. In patients with PH, the volumetric assessment of the RA and the modifications in passive and active emptying fractions, particularly when precedes RV dilation, can become meaningful prognostic markers. These observations underscore the potential of 3D RA assessments in anticipating cardiovascular morbidity and mortality among individuals with PH [84].

**Figure 3:**
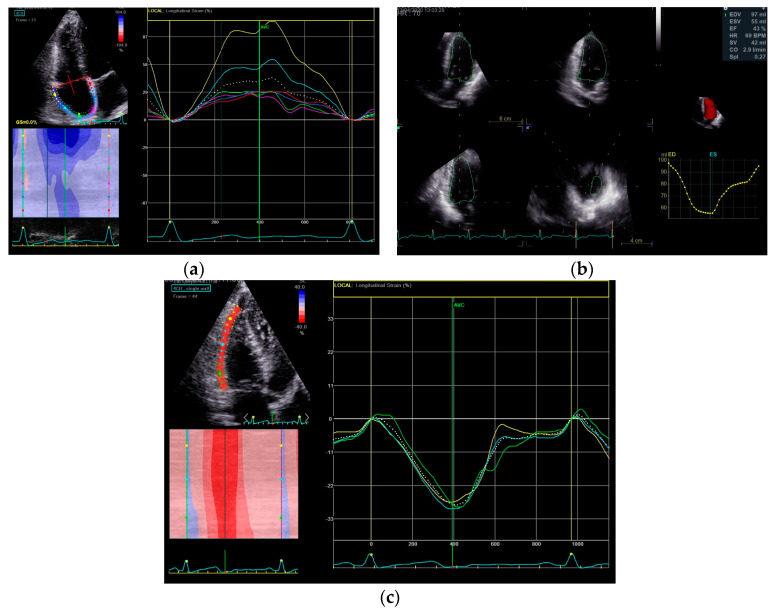
Novel echocardiographic indices of pulmonary hypertension prognosis: (**a**) RA strain; (**b**) 3D RV volume; (**c**) RV strain.

Notably, various aspects of RA phasic function, including reservoir, passive, and active strain, along with RAP, showed correlations with disease severity and overall outcomes [85]. A recent study involving 54 PH patients highlighted the prognostic relevance of RA strain, outlining the significance of RA reservoir function [85]. In a pediatric PH population there was a significant association between RA reservoir function and PASP, PVR, and other systolic parameters (TAPSE, FAC, RV strain), highlighting its prognostic value [86]. Despite those associations, the impact of changes in RA phasic function requires further investigation. Conversely, previous research indicated that impaired RA reservoir function (longitudinal strain) lacks correlation with PASP and PVR [85]. A prospective study of 104 patients with idiopathic PH demonstrated that several factors of RA function, including peak longitudinal strain rate were found to be independently and significantly associated with events and prognosis [67]. Impaired RA reservoir function, as assessed by speckle tracking longitudinal strain, signifies an unfavorable outcome [60]. A prerequisite for 3D echocardiography and strain analysis is the good images quality, which is not always present in patients with PH. Figure 3 provides some representative examples of novel echocardiographic indices for diagnosis and prognosis of PH.

## 6. Conclusions

In conclusion echocardiography remains a reliable, non-invasive, inexpensive, convenient, and easily reproducible modality not only for the preliminary screening of PH but also for PH prognosis. Numerous echocardiographic indices, some of them classical (e.g., TRVpeak and PASP) and some other novel (e.g., RVLS, RA strain) have been proposed for PH diagnosis and prognosis. Their implementation in clinical practice faces significant challenges and the clinicians should be aware of them before echocardiography interpretation. Future studies are required to validate their usage in patients with suspected or established PH.

## Figures and Tables

**Table 1:** Summary of echocardiographic parameters related to pulmonary hypertension diagnosis.

Parameter	Technical Aspects	Correlations with RHC	Limitations
TRVpeak	PSAX, 4Ch, or any modified view. CW doppler through TV	Proportional-moderate to high	Severe TR Assumption of Bernoulli equation
PASP	PSAX, 4Ch, or any modified view. CW doppler through TV	Moderate	Severe TR Assumption of Bernoulli equation Assumption of RAP based on the IVC (size and collapsibility, volume overload situations)—underestimation Rapid equalization of pressures between RA and RV
RVOT-AT	PSAX. May be not well-visualized	High	Arrhythmias Impaired RV function in critically ill patients
Pulmonary regurgitation	PSAX. May be not well-visualized	Low	Rare finding
RV speckle tracking	4Ch view. RV free wall may be not well-visualized	Moderate to high	Limited studies on specific populations Image quality
RA strain	4Ch view may be not well-visualized	No data	Limited studies on specific populations Image quality

4Ch: 4 chamber, CW: continuous wave, IVC: inferior vena cava, PASP: pulmonary arterial systolic pressure, PSAX: parasternal short axis, RA: right atrium, RAP: right atrium pressure, RHC: right heart catheterization, RV: right ventricle, RVOT-AT: right ventricular outflow track acceleration time, TR: tricuspid regurgitation, TRVpeak: tricuspid regurgitation velocity peak.

## Data Availability

The literature cited in this review article was sourced from MEDLINE and EMBASE, Web of Science, Cochrane and Google Scholar databases. All referenced publications are publicly available through these databases, ensuring accessibility and transparency in data availability.
